# Correction: ‘Semen rheology and its relation to male infertility’ (2022), by Tomaiuolo *et al.*

**DOI:** 10.1098/rsfs.2023.0032

**Published:** 2023-12-15

**Authors:** Giovanna Tomaiuolo, Fiammetta Fellico, Valentina Preziosi, Stefano Guido

*Interface Focus* **12**, 20220048 (Published online 6 December 2022). (https://doi.org/10.1098/rsfs.2022.0048)

Regarding figure 8, this was mis-referenced and the caption should state that the figure is adapted from reference [102] Smith DJ, Gaffney E, Gadêlha H, Kapur N, Kirkman-Brown J. 2009 Bend propagation in the flagella of migrating human sperm, and its modulation by viscosity. *Cell Motil. Cytoskeleton* **66**, 220–236. (doi:10.1002/cm.20345) Copyright © 2009, Wiley-Liss.

Regarding reference 47, this was incorrect and the right reference is Sansone A, Di Dato C, de Angelis C *et al*. 2018 Smoke, alcohol and drug addiction and male fertility. *Reprod. Biol. Endocrinol.* **16**, 3. (doi:10.1186/s12958-018-0320-7)

Regarding reference 48, this was incorrect and the right reference is Povey AC, Clyma JA, McNamee R *et al*. 2012 Modifiable and non-modifiable risk factors for poor semen quality: a case-referent study. *Human Reproduction* **27**(9), 2799–2806. (doi:10.1093/humrep/des183)

Regarding reference 75, this was incorrect and the right reference is Fong Neong S, Billington EO, Congly SE. 2018 Sexual dysfunction and sex hormone abnormalities in patients with cirrhosis: review of pathogenesis and management. *Hepatology* **69**, 2683–2695. (doi:10.1002/hep.30359)

Regarding reference 109, this was incorrect and the right reference is Tung CK, Lin C, Harvey B *et al*. 2017 Fluid viscoelasticity promotes collective swimming of sperm. *Sci. Rep.* **7**, 3152. (doi:10.1038/s41598-017-03341-4)

